# Do borderline personality disorder and attention-deficit/hyperactivity disorder co-aggregate in families? A population-based study of 2 million Swedes

**DOI:** 10.1038/s41380-018-0248-5

**Published:** 2018-10-15

**Authors:** Ralf Kuja-Halkola, Kristina Lind Juto, Charlotte Skoglund, Christian Rück, David Mataix-Cols, Ana Pérez-Vigil, Johan Larsson, Clara Hellner, Niklas Långström, Predrag Petrovic, Paul Lichtenstein, Henrik Larsson

**Affiliations:** 1 Department of Medical Epidemiology and Biostatistics, Karolinska Institutet, Stockholm, Sweden; 2 Centre for Psychiatry Research, Department of Clinical Neuroscience, Karolinska Institutet, & Stockholm Health Care Services, Stockholm County Council, Norra Stationsgatan 69, SE-113 64 Stockholm, Sweden; 3 Department of Neuroscience, Uppsala University, Uppsala, Sweden; 4 Department of Clinical Neuroscience, Karolinska Institutet, Stockholm, Sweden; 5 School of Medical Sciences, Örebro University, Örebro, Sweden

**Keywords:** ADHD, Genetics

## Abstract

Large-scale family studies on the co-occurrence of attention-deficit/hyperactivity disorder (ADHD) and borderline personality disorder (BPD) are lacking. Thus, we aimed to estimate the co-occurrence and familial co-aggregation of clinically ascertained ADHD and BPD diagnoses using the entire Swedish population. In a register-based cohort design we included individuals born in Sweden 1979–2001, and identified their diagnoses during 1997–2013; in total, 2,113,902 individuals were included in the analyses. We obtained clinical diagnoses of ADHD and BPD from inpatient and outpatient care. Individuals with an ADHD diagnosis had an adjusted (for birth year, sex, and birth order) odds ratio (aOR) of 19.4 (95% confidence interval [95% CI] = 18.6–20.4) of also having a BPD diagnosis, compared to individuals not diagnosed with ADHD. Having a sibling with ADHD also increased the risk for BPD (monozygotic twins, aOR = 11.2, 95% CI = 3.0–42.2; full siblings, aOR = 2.8, 95% CI = 2.6–3.1; maternal half-siblings, aOR = 1.4, 95% CI = 1.2–1.7; paternal half-siblings, aOR = 1.5, 95% CI = 1.3–1.7). Cousins also had an increased risk. The strength of the association between ADHD and BPD was similar in females and males, and full siblings showed similar increased risks regardless of sex. Among both males and females, ADHD and BPD co-occur within individuals and co-aggregate in relatives; the pattern suggests shared genetic factors and no robust evidence for etiologic sex differences was found. Clinicians should be aware of increased risks for BPD in individuals with ADHD and their relatives, and vice versa.

## Introduction

Attention-deficit/hyperactivity disorder (ADHD) is a common neurodevelopmental disorder [1], with a general population prevalence of 5–10% in childhood [2] and about 2–5% in adulthood [3]. Comorbid disorders associated with ADHD include neurodevelopmental (e.g., autism spectrum disorders), externalizing (e.g., substance use disorders), and internalizing disorders (e.g., depression and anxiety disorders) [4–6]. Borderline personality disorder (BPD) is a common personality disorder, with onset in adolescence, and prevalence in the adult general population of about 1–4% [7–9]. BPD is characterized by emotional dysregulation [10] leading to severely impaired interpersonal functioning, extensive use of health care, and a high risk of suicide [8]. Although it is increasingly recognized that emotional dysregulation is important also in ADHD, population-based data on the co-occurrence of ADHD and BPD within individuals and co-aggregation in families are still lacking [11]. A better understanding of this comorbidity is important. First, it may contribute to the understanding of shared mechanisms underlying both ADHD and BPD [10]. Second, if these disorders share underlying mechanisms, it would suggest that treatment strategies for one disorder may be effective for the other [10].

The association between ADHD and BPD has been addressed in epidemiological studies, suggesting that the disorders often co-occur in the same individuals [11], an association first highlighted in a study of self-reported childhood ADHD symptoms in BPD cases, compared to controls, by Fossati et al. in 2002 [12]. More recently, a Swedish study found that the prevalence of BPD in individuals with ADHD was 37.0% [13], and in a German sample of adult women with BPD, 41.5% were screen-positive for childhood history of ADHD of which 16.1% had ADHD symptoms that persisted into adulthood [14]. A study based on self-reports from approximately 34,000 US individuals found that 33.7% with ADHD also self-reported BPD as compared to 5.2% in the general population [15]. However, as these prior results were mainly based on either small clinical samples or self-reported data, it remains unclear if the co-occurrence of clinically diagnosed ADHD and BPD is present at the population level.

Family and twin studies suggest that susceptibility to ADHD is largely genetic in origin [16–19]. A number of studies have demonstrated genetic overlaps of ADHD with neurodevelopmental, e.g. [5, 20], externalizing, e.g. [21], and internalizing disorders, e.g. [22]^.^ but little is known about the extent to which the genetic risk factors of ADHD are also shared with BPD. Only one twin study has investigated the genetic and environmental contributions to the association between ADHD- and BPD-traits. It showed that half of the association between ADHD and BPD was explained by genetic factors, while the remaining part of the association was explained by environmental factors unique to the individual [23]. Whether these findings generalize to individuals with clinically diagnosed ADHD and BPD and to non-twin samples remains to be investigated.

The main objective of the current study was to estimate the co-occurrence and familial co-aggregation between clinically diagnosed ADHD and BPD in a total population cohort.

## Materials and methods

### Study population

We linked Swedish registers through the unique personal identification number given to each Swedish citizen. The Medical Birth Register [24] provided sex and birth date; we included everyone born between 1 January 1979 and 31 December 2001 (2,319,694 individuals). We excluded stillbirths, congenital malformations, and deaths during infancy (108,079). Using the Cause of Death Register [25], we excluded individuals who died before their 12th birthday (6283). Via the Total Population Register [26] we excluded individuals who emigrated before their 12th birthday (72,337). Age 12 represents the latest age of first presentation of ADHD symptoms according to DSM-5. Finally, using the Multi-Generation Register [27], we linked individuals to their biological parents, and excluded individuals who did not have both parents known (19,093), yielding a sample of 2,113,902 individuals. The Multi-Generation Register and the Twin Register [28] allowed us to identify relatives: monozygotic twins (sharing essentially 100% of alleles), dizygotic twins (sharing on average 50% of co-segregating alleles), full siblings (50% of co-segregating alleles), maternal half-siblings (25%), paternal half-siblings (25%); cousins whose parents were full siblings (12.5%); cousins whose parents were maternal- and paternal half-siblings (6.25%).

The study was approved by the Regional Ethical Review Board in Stockholm (Dnr 2013/862–31/5); since it was a registry study no individual was contacted, and informed consent was waived. The validation sub-study was approved by the Swedish Central Ethics Board (Dnr Ö 27-2012).

### Study variables

We linked the data to the Swedish National Patient Register [29] (NPR) and Prescribed Drug Register [30] (PDR). The NPR comprises diagnoses from inpatient health care, and from outpatient visits to specialist care from 2001 and onwards [29]; we used International Classification of Diseases 10th revision [31] (ICD-10) diagnoses from 1 January 1997 (when ICD-10 was introduced in Sweden) to 31 December 2013. We used data from PDR on all dispensed medications from 1 June 2005 to 31 December 2014.

We identified individuals with ADHD from the NPR (ICD-10 code F90; Hyperkinetic Disorder) and/or from the PDR via prescriptions of ADHD medications (methylphenidate, amphetamine, dexamphetamine, lisdexamfetamine, or atomoxetine). Details supporting the validity of this definition are available in previous publications, including the validity of using ADHD medications as proxy for ADHD diagnosis [32, 33].

We identified individuals with BPD from the NPR (ICD-10 code F60.3; Emotionally Unstable Personality Disorder, the diagnosis code used in Swedish version of ICD-10 to correspond to DSM-IV-TR Borderline Personality Disorder) [34]. As the validity of the BPD diagnosis in the NPR had not been previously investigated, we performed a separate validation study, following a previously described methodology [35]. Briefly, we requested 100 randomly selected medical records for men and women with a BPD diagnosis in the NPR. Two clinically experienced, board-certified, adult psychiatrists independently examined the records employing DSM-IV-TR (very similar to the ICD-10 research criteria) when assessing diagnosis in the records. Evidence of diagnosis was considered to be present if (a) at least five of the nine criteria in BPD were endorsed in the record or whether a diagnostic instrument (SCID-II) had been employed to establish a diagnosis of BPD, or (b) the clinicians held an expert opinion that a BPD diagnosis was the most likely explanation for the patients symptoms given the information available. We received 82 of the requested records, and 70 had sufficient information to assess diagnosis. The agreement for endorsed diagnosis between examiners was good (93%; 95% confidence interval [95% CI], 84–98) as was the inter-rater reliability for number of endorsed criteria (Cohens kappa [36], 0.82; 95% CI, 0.74–0.90). Disagreements on endorsing diagnosis (5 records) were solved by using the least favorable rater (i.e., if one rated as “not BPD” then her rating had precedence). According to (a) 44 diagnoses could be confirmed, with the addition of (b) this number increased to 57 records, corresponding to positive predictive values (PPV) of 63% (95% CI = 50–74) and 81% (95% CI = 70–90).

Sex, birth year, and birth order are collectively referred to as the covariates below—included since they are potential confounders, associated with both ADHD and BPD. Birth year was included as a covariate to adjust for the secular changes affecting diagnostic practices through the follow-up period; for instance, incidence of ADHD diagnoses has seen a sharp increase. Birth order was included because the position among siblings may affect the likelihood of getting diagnoses.

### Statistical analyses

We estimated associations using logistic regression, thus viewing ADHD and BPD as binary variables. The choice of logistic regression, even though age at diagnosis is accessible to us, reflects that we do not believe the date of diagnosis accurately captures the date of disease onset. Additionally this means that analyses of temporal order of diagnoses could be misleading. To estimate appropriate associations we instead rely on a carefully selected cohort with reasonable follow-up, adjustment for birth year, and sensitivity analyses.

#### Co-occurrence within individuals

We analyzed the within-individual association between ADHD (exposure) and BPD (outcome) to obtain crude and covariate-adjusted odds ratios (OR and aOR). Note though that the choice of which of diagnoses to view as exposure and outcome is arbitrary in the current design. We calculated proportions of BPD among those with and without ADHD in the total cohort and separately by sex (crudely and standardized over covariates) [37], and the corresponding proportions of ADHD in BPD.

#### Co-aggregation within relatives

We estimated the risk of BPD (outcome) in individuals according to their relatives’ ADHD (exposure): crude and adjusted for covariates, including covariates for the relative. Similarly as in the within-individual analyses the choice of exposure and outcome is arbitrary. Comparing estimates across relatives informs about the role of genetic and/or environmental risk factors shared by the disorders. If the association between monozygotic twins is weaker than the within-individual association, then factors not shared by family members are likely to influence the co-occurrence. If the association between full siblings is weaker than between monozygotic twins, and the association between maternal half-siblings is weaker than between full siblings, genetic factors are likely to influence the association. Maternal half-siblings have similar intra-uterine environment, and usually grow up in similar environments since children predominantly stay with the mother following parental separation [38, 39]. Hence, a stronger association in maternal than paternal half-siblings suggest shared environment.

Further, we calculated ORs in the full sibling subsample stratified by sex combinations, and calculated proportions with BPD diagnosis. We calculated similar proportions using ADHD as outcome and BPD as exposure.

All analyses were performed in R, adjusting the precision of estimates for dependencies within family clusters using cluster-robust sandwich estimators, employing the packages drgee [40] and stdReg [37].

### Sensitivity analyses

#### Alternative diagnostic definitions

The main cohort covered many birth years to provide reasonable power for both the within individual and the relative analyses, which introduced a potential risk of bias due to both left truncation (i.e., we started following some birth cohorts later in life) and right censoring (i.e., individuals were not followed until death). To investigate if results were influenced by this we identified a sub-cohort where BPD was defined as present if an individual was eligible to get a diagnosis through high-risk age period from age 18 until at least age 23, taking migration and deaths into account, and was diagnosed with BPD at any time (including before age 18 and after age 23). If the individual did not have an uninterrupted period with possibility to get a diagnosis between ages 18 and 23—due to end of follow-up, not being alive, or not living in Sweden—we defined BPD as missing; else we defined it as “no BPD”. We defined ADHD correspondingly, with safeguarded observation from age 7 until age 12. Using these definitions, we investigated the robustness of associations.

#### Test of familial factors

Between full siblings, we adjusted analyses for ADHD occurrence in the outcome individual, yielding estimates not interpretable as familial risks since the adjustment introduces bias. However, this analysis helps determine whether a shared familial cause (e.g., genetic) for an association likely exists, regardless of if one disease causes the other directly. If the association remains despite such adjustment, this supports the notion of a liability for both disorders shared by relatives (see, e.g., [41] for further explanation).

## Results

### Co-occurrence within individuals

During follow-up 82,593 (3.9%) individuals were diagnosed with ADHD, while 9544 (0.5%) were diagnosed with BPD (Table 1). Males were more frequently diagnosed with ADHD, while BPD diagnoses were more common among females. Among individuals with an ADHD diagnosis, 3.6% had also been diagnosed with BPD (2952 out of 82,593; Table 2), with a higher proportion in females (8.0%) compared to males (1.0%; Table 2). Among individuals with a BPD diagnosis, 30.9% had also been diagnosed with ADHD (2952 out of 9544), with a higher proportion in males (40.1%) compared to females (29.6%; Table 2). Calculating proportions standardized over covariates suggest that the increase was not due to a skewed age- and/or sex distribution (Supplemental Table 1a and c). Having an ADHD diagnosis increased the odds of a BPD diagnosis 11.4 times (95% CI = 10.9–11.9; Table 3), and even more so after adjustment for covariates (aOR = 19.4, 95% CI = 18.6–20.4; Table 3). In Supplemental Table 2 separate adjustment for each covariate are presented; both for within individual and relatives analyses adjustment for birth year had the largest impact on the estimates. The association was stronger in women compared to men (aOR = 19.1 vs. 21.8; *p* = 0.047; Table 4), but this difference did not remain statistically significant in sensitivity analysis with observation ensured in high-risk periods (*p* = 0.720; Supplemental Table 3).

**Table 1:** Descriptive information

	Total sample *N* (column percent)	ADHD *N* (row percent)	BPD *N* (row percent)	ADHD and BPD *N* (row percent)
Sample	2,113,902 (100%)	82,593 (3.9%)	9544 (0.5%)	2952 (0.1%)
Covariates				
*Sex*				
Female	1,031,987 (48.8%)	30,571 (3.0%)	8307 (0.8%)	2456 (0.2%)
Male	1,081,915 (51.2%)	52,022 (4.8%)	1237 (0.1%)	496 (<0.1%)
*Birth years*				
1979–1984	505,457 (23.9%)	10,763 (2.1%)	3761 (0.7%)	1091 (0.2%)
1985–1989	483,733 (22.9%)	13,989 (2.9%)	3601 (0.7%)	1102 (0.2%)
1990–1994	543,344 (25.7%)	24,095 (4.4%)	2079 (0.4%)	711 (0.1%)
1995–2001	581,368 (27.5%)	33,746 (5.8%)	103 (<0.1%)	48 (<0.1%)
*Birth order by mother*				
1	874,369 (41.4%)	35,204 (4.0%)	4179 (0.5%)	1316 (0.2%)
2	772,164 (36.5%)	28,406 (3.7%)	3143 (0.4%)	974 (0.1%)
3	332,840 (15.7%)	12,512 (3.8%)	1505 (0.5%)	422 (0.1%)
4	94,430 (4.5%)	4380 (4.6%)	504 (0.5%)	159 (0.2%)
5	26,228 (1.2%)	1398 (5.3%)	140 (0.5%)	57 (0.2%)
>5	13,873 (0.7%)	693 (5.0%)	73 (0.5%)	24 (0.2%)

**Table 2:** Proportion of BPD and ADHD in individuals with the other diagnosis, and in individuals whose relatives have the other diagnosis

	No. of individuals	Proportion of BPD—outcome Percent (95% CI)		Proportion of ADHD—outcome Percent (95% CI)	
		No ADHD	ADHD	No BPD	BPD
*Within individual*					
Both sexes^a^	2,113,902	0.3% (0.3–0.3)	3.6% (3.4–3.7)	3.8% (3.8–3.8)	30.9% (30.0–31.9)
Females only^a^	1,031,987	0.6% (0.6–0.6)	8.0% (7.7–8.3)	2.7% (2.7–2.8)	29.6% (28.6–30.5)
Males only^a^	1,081,915	0.1% (0.1–0.1)	1.0% (0.9–1.0)	4.8% (4.7–4.8)	40.1% (37.4–42.8)
*Full siblings*	*No. of pairs*^b^				
Both sexes	2,211,396	0.4% (0.4–0.4)	0.9% (0.9–1.0)	3.4% (3.4–3.4)	7.8% (7.2–8.4)
Female outcome female exposure	523,464	0.7% (0.7–0.7)	1.9% (1.6–2.1)	2.6% (2.5–2.6)	6.6% (5.8–7.4)
Female outcome male exposure	552,516	0.7% (0.7–0.8)	1.5% (1.3–1.7)	2.6% (2.6–2.7)	8.6% (6.2–11.0)
Male outcome female exposure	552,516	0.1% (0.1–0.1)	0.3% (0.2–0.4)	4.2% (4.2–4.3)	8.4% (7.6–9.3)
Male outcome male exposure	582,900	0.1% (0.1–0.1)	0.2% (0.2–0.3)	4.1% (4.0–4.2)	10.6% (7.9–13.3)
*Half-siblings*	*Pairs*^b^				
Both maternal and paternal	663,566	0.9% (0.8–0.9)	1.3% (1.2–1.3)	7.7% (7.6–7.8)	10.6% (9.9–11.4)

Notes: *95% CI* 95% confidence intervals

^a^Cluster-robust standard errors based on mothers as clusters

^b^Number of unique ways of combining pairs, i.e., a pair may be included twice, first with A as outcome person and B as exposure person, then with B as outcome person and A as exposure person

**Table 3:** Odds ratio of a BPD diagnosis when having an ADHD diagnosis oneself, or a relative diagnosed with ADHD

	No. of individuals	Crude odds ratio (95% CI)	Adjusted odds ratio^a^ (95% CI)
Within individual^b^	2,113,902	11.4 (10.9–11.9)	19.4 (18.6–20.4)
*Relatives*	*No. of pairs*^c^		
Monozygotic twins	9130	5.0 (1.5–16.9)	11.2 (3.0–42.2)
Dizygotic twins	17,350	0.6 (0.1–4.7)	1.0 (0.1–7.3)
Full siblings	2,211,396	2.4 (2.2–2.6)	2.8 (2.6–3.1)
Maternal half-siblings	332,486	1.4 (1.2–1.6)	1.4 (1.2–1.7)
Paternal half-siblings	331,080	1.5 (1.3–1.6)	1.5 (1.3–1.7)
Cousins parents full siblings	6,456,848	1.4 (1.3–1.5)	1.5 (1.4–1.6)
Cousins parents maternal half-siblings	472,212	1.2 (1.0–1.4)	1.3 (1.1–1.5)
Cousins parents paternal half-siblings	466,836	1.2 (1.0–1.4)	1.2 (1.0–1.4)

Notes: *95% CI* 95% confidence intervals

^a^Adjusted for sex, sex of relative, birth year, birth year of relative, birth order, and birth order of relative, wherever applicable

^b^Cluster-robust standard errors based on mothers as clusters

^c^Number of unique ways of combining pairs, i.e., a pair may be included twice, first with A as outcome person and B as exposure person, then with B as outcome person and A as exposure person

**Table 4:** Odds ratio of a BPD diagnosis when having an ADHD diagnosis oneself, or a full sibling diagnosed with ADHD. Sex-specific analyses

	No. of individuals	Crude odds ratio (95% CI)	Adjusted odds ratio^a^ (95% CI)	*p*-Value^b^
Within females^c^	1,031,987	14.9 (14.2–15.6)	19.1 (18.2–20.1)	0.047
Within males^c^	1,081,915	13.4 (11.9–15.0)	21.8 (19.3–24.5)	
*Full siblings*	*No. of pairs*^d^			
Female outcome female exposure	523,464	2.7 (2.3–3.1)	2.9 (2.6–3.4)	0.087
Female outcome male exposure	552,516	2.1 (1.9–2.3)	2.5 (2.3–2.8)	
Male outcome female exposure	552,516	3.5 (2.6–4.7)	3.9 (2.9–5.4)	0.683
Male outcome male exposure	582,900	2.8 (2.1–3.7)	3.6 (2.7–4.8)	

Notes: *95% CI* 95% confidence intervals

^a^Adjusted for birth year, birth year of relative, birth order, and birth order of relative, where applicable

^b^For pairwise comparison of adjusted estimates

^c^Cluster-robust standard errors based on mothers as clusters

^d^Number of unique ways of combining pairs, i.e., a pair may be included twice, first with A as outcome person and B as exposure person, then with B as outcome person and A as exposure person

### Co-aggregation within relatives

Having a relative with an ADHD diagnosis increased the odds of a BPD diagnosis (Table 3). The aOR for monozygotic twins (11.2, 95% CI = 3.0–42.2) was lower than within individuals, which indicate that factors not shared by family members are likely to influence the co-occurrence. The aOR for dizygotic twins was 1.0, with a wide CI (95% CI = 0.1–7.3)—reflecting the low power for these relatives. Among full siblings, the aOR was 2.8 (95% CI = 2.6–3.1), significantly lower compared to monozygotic twins (*p* = 0.041), suggesting that at least part of the association between BPD and ADHD is explained by genetic factors. The aORs were lower in both maternal (1.4, 95% CI = 1.2–1.7) and paternal half-siblings (1.5, 95% CI = 1.3–1.7) compared to full siblings (both *p* < 0.001), again supporting genetic contributions to the association. Further, maternal and paternal half-sibling estimates did not differ significantly from each other (*p* = 0.671), indicating that shared environmental effects have little or no impact on the association. Cousins whose parents were full siblings had an increased risk of having a diagnosis of BPD, aOR, 1.5 (95% CI = 1.4–1.6), while cousins whose parents were half-siblings had even lower aOR.

If etiological sex differences were present (e.g., partly different genetic factors being responsible for the overlap between the disorders in males and females) the increased risk for BPD would be expected to differ depending on sex. That is, a same-sex sibling with ADHD should increase the risk for BPD more compared to having an opposite-sex sibling with ADHD. Further, it has been suggested that females’ ADHD diagnoses possibly represent a stronger genetic load compared to males [42]. If this genetic load was also associated with BPD, siblings of females with ADHD should have an increased risk of BPD compared to siblings of males with ADHD. In full siblings, females having a sister with ADHD had an increased risk of having a BPD diagnosis compared to females having a brother with ADHD (aOR = 2.9 vs. 2.5; Table 4). Males with a sister diagnosed with ADHD also exhibited increased risk compared to males with a brother with ADHD (aOR = 3.9 vs 3.6; Table 4). However, neither of these differences were statistically significant (*p* = 0.087 in females and *p* = 0.683 in males; Table 4). Thus, etiological sex differences were not robustly supported.

### Sensitivity analyses

#### Alternative diagnostic definitions

Analyses safeguarding follow-up during high-risk periods in ADHD and BPD revealed similar patterns of proportions of diagnoses, although with slightly higher percentages, 5.1% for ADHD and 0.7% for BPD (Supplemental Table 4). Analyses suggested ORs largely similar to those found in the main analysis, both within individuals and relatives (Supplemental Table 3). Corresponding results for proportion with diagnoses also followed the same pattern as seen with the original diagnostic definitions (Supplemental Table 1b and d).

#### Test of familial factors

Analyses of full siblings with additional adjustment of ADHD in the outcome person found OR significantly higher than 1 (aOR = 1.4, 95% CI = 1.2–1.5). Altogether, findings support that familial factors are partly responsible for the observed co-aggregation within relatives, and not an artifact of one disorder causing the other within individuals.

## Discussion

This is the largest study of ADHD and BPD co-occurrence, in a total population of over 2.1 million individuals, and the only one exploring familial co-aggregation of clinically diagnosed ADHD and BPD. We found that individuals with a diagnosis of ADHD had a 19.4 times higher odds of BPD diagnosis than individuals not diagnosed with ADHD. Furthering a previous twin study on the relation between ADHD- and BPD-like traits [23], the pattern of familial co-aggregation of ADHD and BPD across different types of relatives indicated that genetic factors play a role in ADHD and BPD co-occurrence. Our results should contribute to improved understanding of the mechanisms underlying both ADHD and BPD [10].

Emotional dysregulation has since long been hypothesized to underlie BPD, but also suggested to be an important aspect of ADHD [43–45]. This is in line with our results indicating that the shared genetic origin of ADHD and BPD may reflect a load of emotional dysregulation processes involved in both disorders, that the processes underlying the disorders are separate but correlated, or a combination thereof [10].

Although genetic factors were implied, the estimated associations between siblings compared to within individuals indicated that not all of the association was explained by these factors. Similarly to a previous study [23], our results indicate that individually unique factors, including a potential direct (phenotypic/causal) effect of ADHD diagnosis on the probability of getting a BPD diagnosis, are likely to explain parts of the association.

We found that BPD was more common among females, while ADHD was more so among males. In contrast, the data supported no, or only minor, differences in etiological factors responsible for the observed associations between ADHD and BPD in males and females. This may suggest that differences in the co-occurrence rates between males and females are probably explained by other factors, such as detection bias or diagnostic traditions—supported by lack evidence of sex differences in prevalence when assessed by face-to-face interviews of adults in the US [46].

Limitations of this study include under-detection; the observed diagnoses are likely underestimations of the true proportion of individuals with symptom levels and impairments corresponding to diagnoses if assessed by healthcare professionals. Some individuals may have a diagnosis registered in sources not accessible by us, e.g. outpatient care prior to 2001, or just do not seek help. Under-detection would be likely to bias estimates towards null. Since ADHD and BPD are likely to suffer from different detection biases—including different likelihood of detection depending on during which ages the individuals were followed up in available registries—we performed sensitivity analyses in a subsample with better coverage in high-risk periods for both diagnoses; no major differences in results appeared (Supplemental Table 3). Another limitation, likely to bias estimates upwards, is correlated detection bias; individuals with a diagnosis, or their relatives, will be in closer contact with health care and therefore have a higher probability of receiving another diagnosis compared to individuals with similar symptom- and impairment levels without other diagnoses. Another potential source of bias is misdiagnosis, where, for instance, BPD may be misdiagnosed as ADHD by a clinician and later more correctly diagnosed as BPD, thus introducing a spurious association between the disorders. Such diagnostic issues are plausible because of overlaps in the diagnostic symptom criteria between ADHD and BPD [11]. Another limitation is that the register diagnoses used are set by clinicians without enforced standardized procedures in place. This may increase the heterogeneity of symptom profiles sharing the same diagnosis and are likely to result in less specificity of diagnoses. However, it also represents a more naturalistic result of the relation between diagnoses given in the clinical environment. For our ADHD-definition we did not include ICD-10 diagnosis F98.8—“Other specified behavioral and emotional disorders with onset usually occurring in childhood and adolescence”—which include “Attention-deficit disorder without hyperactivity” (ADD). This because of inability to discern this important sub-diagnosis from other sub-diagnoses (i.e., excessive masturbation, nail-biting, nose-picking, and thumb-sucking), as well as it being questionable if this sub-diagnosis is consistently used for ADD in concurrent clinical practice. Thus, we may be missing to identify some individuals with ADD, while avoiding incorrectly identifying individuals without ADHD problems as having ADHD diagnosis. However, in our data, only 739 individuals had F98.8 without ADHD according to our definition, and a post-hoc analysis including F98.8 did not change our results (Supplemental Table 5). We did not follow individuals throughout their lives, thus they were at risk of receiving a diagnosis outside the follow-up period. However, when enforcing follow-up through high-risk periods for ADHD and BPD, associations remained robust.

Important strengths of the current study are its population-based cohort, sample size, and clinically diagnosed ADHD and BPD. Although both ADHD and BPD are likely to include heterogeneous presentations, the diagnoses are likely to capture the DSM-IV-criteria accurately—the validation of the ICD-10 BPD codes showed adequate agreement (93%) and acceptable precision (PPV = 81%). A further strength is the use of population registers, where data are gathered prospectively and primarily for administrative purposes, and, importantly, totally independently from the current study.

ADHD and BPD co-occur in individuals, and co-aggregate in relatives—reflecting both shared genetic risk factors and individually unique risk factors. Although ADHD is more common in males and BPD in females, the strength of the individual and familial associations between the disorders is similar across sexes, indicating that etiologic sex differences are unlikely. Clinicians need to be aware of increased risk for BPD in individuals with a diagnosis of ADHD, as well as in their relatives, and vice versa.

## Disclaimer

The funders had no role in the design and conduct of the study; collection, management, analysis, and interpretation of the data; preparation, review, or approval of the manuscript; and decision to submit the manuscript for publication.

## Electronic supplementary material

Supplemental online material
