# Supplementary material for: Do borderline personality disorder and attention-deficit/hyperactivity disorder co-aggregate in families? A population-based study of 2 million Swedes
Source: Mol Psychiatry. 2018 Oct 15;26(1):341–9. doi: 10.1038/s41380-018-0248-5 (PMC7815504; doi:10.1038/s41380-018-0248-5)
Supplement: Supplementary file 1 — Supplemental online material [file 41380_2018_248_MOESM1_ESM.docx]

# Do Borderline Personality Disorder and Attention-Deficit/Hyperactivity Disorder co-aggregate in families? A population-based study of 2 million Swedes

Ralf Kuja-Halkola, PhD^1^; Kristina Lind Juto, MD^1^; Charlotte Skoglund, MD, PhD^2^; Christian Rück, MD, PhD^2^; David Mataix-Cols, MD, PhD^2^; Ana Pérez-Vigil, MD^2^; Johan Larsson, MD^2^; Clara Hellner, MD, PhD^2^; Niklas Långström, MD, PhD^3^; Predrag Petrovic, MD, PhD^4^; Paul Lichtenstein, PhD^1^; and Henrik Larsson, PhD^1,5^

**Author Affiliations:**
^1^ Department of Medical Epidemiology and Biostatistics, Karolinska Institutet, Stockholm, Sweden
^2^ Centre for Psychiatry Research, Department of Clinical Neuroscience, Karolinska Institutet, Stockholm, Sweden
^3^ Department of Neuroscience, Uppsala University, Uppsala, Sweden
^4^ Department of Clinical Neuroscience, Karolinska Institutet, Stockholm, Sweden
^5^ School of Medical Sciences, Örebro University, Örebro, Sweden

**Corresponding author:** Ralf Kuja-Halkola, Department of Medical Epidemiology and Biostatistics, Karolinska Institutet, PO Box 281, SE-171 77 Stockholm, Sweden ([ralf.kuja-halkola@ki.se](mailto:ralf.kuja-halkola@ki.se))

**Supplemental Table 1a**. Proportion of BPD and ADHD in individuals with the other diagnosis, and in individuals whose relatives have the other diagnosis. Estimates are crude and standardized over covariates.

| **BPD as outcome** |  |  |  |  |  |  |  |  |
| --- | --- | --- | --- | --- | --- | --- | --- | --- |
|  | **Crude**  **(95% CI)** |  |  |  | **Adjusted**^1^  **(95% CI)** |  |  |  |
|  | **No ADHD** | **ADHD** |  |  | **No ADHD** | **ADHD** |  |  |
| Within individuals | 0.3% (0.3-0.3) | 3.6% (3.4-3.7) |  |  | 0.3% (0.3-0.3) | 5.4% (5.3-5.6) |  |  |
| Within females | 0.6% (0.6-0.6) | 8.0% (7.7-8.3) |  |  | 0.6% (0.6-0.6) | 9.6% (9.3-10.0) |  |  |
| Within males | 0.1% (0.1-0.1) | 1.0% (0.9-1.0) |  |  | 0.1% (0.1-0.1) | 1.5% (1.4-1.6) |  |  |
| **Full siblings** | **No ADHD or BPD** | **ADHD but no BPD** | **BPD but no ADHD** | **ADHD and BPD** | **No ADHD or BPD** | **ADHD but no BPD** | **BPD but no ADHD** | **ADHD and BPD** |
| Both sexes | 0.4% (0.4-0.4) | 0.9% (0.8-1.0) | 2.1% (1.7-2.6) | 2.6% (1.9-3.3) | 0.4% (0.4-0.4) | 1.0% (0.9-1.1) | 1.7% (1.3-2.1) | 2.1% (1.5-2.7) |
| Female outcome, female exposure | 0.7% (0.7-0.7) | 1.7% (1.4-1.9) | 3.9% (3.0-4.8) | 4.5% (3.0-6.0) | 0.7% (0.7-0.7) | 1.9% (1.6-2.1) | 3.1% (2.4-3.9) | 3.5% (2.4-4.7) |
| Female outcome, male exposure | 0.7% (0.7-0.8) | 1.5% (1.3-1.6) | 2.7% (1.0-4.4) | 5.9% (2.8-8.9) | 0.7% (0.7-0.7) | 1.8% (1.6-1.9) | 2.1% (0.8-3.5) | 4.6% (2.2-6.9) |
| Male outcome, female exposure | 0.1% (0.1-0.1) | 0.3% (0.2-0.4) | 0.4% (0.2-0.7) | 0.8% (0.3-1.3) | 0.1% (0.1-0.1) | 0.3% (0.2-0.4) | 0.3% (0.2-0.5) | 0.6% (0.2-1.0) |
| Male outcome, male exposure | 0.1% (0.1-0.1) | 0.2% (0.2-0.3) | 1.5% (-0.2-3.2) | 0.4% (-0.4-1.3) | 0.1% (0.1-0.1) | 0.3% (0.2-0.4) | 1.0% (-0.2-2.2) | 0.3% (-0.3-0.9) |

Notes: 95% CI, 95% confidence intervals.
^1^ Adjusted for covariates sex, sex of relative, birth year, birth year of relative, birth order, and birth order of relative, where applicable. Proportions are standardized over covariates.**Supplemental Table 1b**. Proportion of BPD and ADHD in individuals with the other diagnosis, and in individuals whose relatives have the other diagnosis. Estimates are crude and standardized over covariates. Sensitivity analysis with ADHD and BPD defined more restrictively.

| **BPD as outcome** |  |  |  |  |  |  |  |  |
| --- | --- | --- | --- | --- | --- | --- | --- | --- |
|  | **Crude**  **(95% CI)** |  |  |  | **Adjusted**^1^  **(95% CI)** |  |  |  |
|  | **No ADHD** | **ADHD** |  |  | **No ADHD** | **ADHD** |  |  |
| Within individuals | 0.4% (0.3-0.4) | 4.3% (3.9-4.7) |  |  | 0.4% (0.3-0.4) | 5.1% (4.6-5.6) |  |  |
| Within females | 0.7% (0.6-0.7) | 9.5% (8.5-10.4) |  |  | 0.7% (0.6-0.7) | 9.4% (8.4-10.4) |  |  |
| Within males | 0.1% (0.0-0.1) | 0.9% (0.7-1.2) |  |  | 0.1% (0.0-0.1) | 0.9% (0.7-1.2) |  |  |
| **Full siblings** | **No ADHD or BPD** | **ADHD but no BPD** | **BPD but no ADHD** | **ADHD and BPD** | **No ADHD or BPD** | **ADHD but no BPD** | **BPD but no ADHD** | **ADHD and BPD** |
| Both sexes | 0.6% (0.6-0.7) | 1.8% (1.4-2.2) | 1.5% (0.3-2.8) | 4.2% (1.2-7.1) | 0.6% (0.6-0.7) | 1.8% (1.4-2.2) | 1.5% (0.3-2.6) | 4.0% (1.2-6.9) |
| Female outcome, female exposure | 1.2% (1.1-1.3) | 2.8% (1.7-3.9) | 3.4% (0.8-6.0) | 7.9% (1.6-14.2) | 1.2% (1.1-1.3) | 2.8% (1.7-3.9) | 3.3% (0.8-5.9) | 7.6% (1.3-13.8) |
| Female outcome, male exposure | 1.1% (1.0-1.2) | 3.3% (2.4-4.2) | 0.0% (0.0-0.0) | 11.8% (-3.8-27.3) | 1.1% (1.0-1.2) | 3.3% (2.4-4.3) | 0.0% (0.0-0.0) | 12.6% (-3.8-29.1) |
| Male outcome, female exposure | 0.1% (0.1-0.2) | 0.6% (0.1-1.2) | 0.0% (0.0-0.0) | 0.0% (0.0-0.0) | 0.1% (0.1-0.2) | 0.6% (0.1-1.2) | 0.0% (0.0-0.0) | 0.0% (0.0-0.0) |
| Male outcome, male exposure | 0.1% (0.1-0.2) | 0.4% (0.1-0.8) | 0.0% (0.0-0.0) | 0.0% (0.0-0.0) | 0.1% (0.1-0.2) | 0.4% (0.1-0.7) | 0.0% (0.0-0.0) | 0.0% (0.0-0.0) |

Notes: 95% CI, 95% confidence intervals.
^1^ Adjusted for covariates sex, sex of relative, birth year, birth year of relative, birth order, and birth order of relative, where applicable. Proportions are standardized over covariates.

**Supplemental Table 1c**. Proportion of BPD and ADHD in individuals with the other diagnosis, and in individuals whose relatives have the other diagnosis. Estimates are crude and standardized over covariates.

| **ADHD as outcome** |  |  |  |  |  |  |  |  |
| --- | --- | --- | --- | --- | --- | --- | --- | --- |
|  | **Crude**  **(95% CI)** |  |  |  | **Adjusted**^1^  **(95% CI)** |  |  |  |
|  | **No BPD** | **BPD** |  |  | **No BPD** | **BPD** |  |  |
| Within individuals | 3.8% (3.8-3.8) | 30.9% (30.0-31.9) |  |  | 3.8% (3.7-3.8) | 42.4% (41.3-43.4) |  |  |
| Within females | 2.7% (2.7-2.8) | 29.6% (28.6-30.5) |  |  | 2.7% (2.7-2.8) | 34.2% (33.1-35.2) |  |  |
| Within males | 4.8% (4.7-4.8) | 40.1% (37.4-42.8) |  |  | 4.8% (4.7-4.8) | 49.5% (46.8-52.3) |  |  |
| **Full siblings** | **No BPD or ADHD** | **BPD but no ADHD** | **ADHD but no BPD** | **BPD and ADHD** | **No BPD or ADHD** | **BPD but no ADHD** | **ADHD but no BPD** | **BPD and ADHD** |
| Both sexes | 2.9% (2.9-3.0) | 5.7% (5.1-6.3) | 16.9% (16.5-17.3) | 12.9% (11.6-14.3) | 3.0% (2.9-3.0) | 6.3% (5.6-6.9) | 15.9% (15.5-16.3) | 13.8% (12.3-15.2) |
| Female outcome, female exposure | 2.3% (2.2-2.3) | 4.9% (4.1-5.7) | 13.7% (12.9-14.6) | 11.4% (9.4-13.4) | 2.3% (2.2-2.3) | 5.2% (4.4-6.1) | 13.2% (12.3-14.0) | 12.2% (10.1-14.4) |
| Female outcome, male exposure | 2.2% (2.1-2.2) | 4.5% (2.3-6.7) | 12.9% (12.4-13.4) | 14.9% (10.1-19.6) | 2.2% (2.1-2.2) | 4.8% (2.4-7.1) | 12.3% (11.9-12.8) | 15.7% (10.8-20.6) |
| Male outcome, female exposure | 3.8% (3.7-3.9) | 6.4% (5.5-7.3) | 21.6% (20.9-22.4) | 13.6% (11.7-15.6) | 3.8% (3.7-3.9) | 7.5% (6.5-8.6) | 20.1% (19.4-20.8) | 15.3% (13.1-17.5) |
| Male outcome, male exposure | 3.4% (3.4-3.5) | 8.2% (5.1-11.2) | 19.8% (19.1-20.6) | 14.1% (9.3-18.9) | 3.5% (3.4-3.5) | 9.9% (6.3-13.5) | 17.9% (17.2-18.6) | 16.2% (10.8-21.6) |

Notes: 95% CI, 95% confidence intervals.
^1^ Adjusted for covariates sex, sex of relative, birth year, birth year of relative, birth order, and birth order of relative, where applicable. Proportions are standardized over covariates.**Supplemental Table 1d**. Proportion of BPD and ADHD in individuals with the other diagnosis, and in individuals whose relatives have the other diagnosis. Estimates are crude and standardized over covariates. Sensitivity analysis with ADHD and BPD defined more restrictively.

| **ADHD as outcome** |  |  |  |  |  |  |  |  |
| --- | --- | --- | --- | --- | --- | --- | --- | --- |
|  | **Crude**  **(95% CI)** |  |  |  | **Adjusted**^1^  **(95% CI)** |  |  |  |
|  | **No BPD** | **BPD** |  |  | **No BPD** | **BPD** |  |  |
| Within individuals | 3.7% (3.6-3.8) | 32.1% (29.4-34.8) |  |  | 3.7% (3.6-3.8) | 36.4% (33.5-39.3) |  |  |
| Within females | 2.9% (2.8-3.0) | 30.9% (28.1-33.7) |  |  | 2.9% (2.8-3.0) | 30.8% (28.0-33.7) |  |  |
| Within males | 4.5% (4.4-4.6) | 43.2% (34.0-52.5) |  |  | 4.5% (4.4-4.6) | 43.2% (33.9-52.5) |  |  |
| **Full siblings** | **No BPD or ADHD** | **BPD but no ADHD** | **ADHD but no BPD** | **BPD and ADHD** | **No BPD or ADHD** | **BPD but no ADHD** | **ADHD but no BPD** | **BPD and ADHD** |
| Both sexes | 4.1% (4.0-4.3) | 8.0% (5.4-10.5) | 20.0% (18.7-21.3) | 22.3% (15.5-29.0) | 4.1% (4.0-4.3) | 7.8% (5.3-10.3) | 20.1% (18.8-21.5) | 21.1% (14.8-27.5) |
| Female outcome, female exposure | 3.2% (3.0-3.4) | 8.3% (4.6-12.0) | 15.8% (13.0-18.7) | 16.1% (6.7-25.5) | 3.2% (3.0-3.4) | 8.3% (4.6-12.0) | 15.8% (12.9-18.6) | 16.2% (6.8-25.5) |
| Female outcome, male exposure | 3.0% (2.8-3.2) | 0.0% (0.0-0.0) | 15.4% (13.5-17.3) | 8.3% (-7.4-24.1) | 3.0% (2.8-3.2) | 0.0% (0.0-0.0) | 15.3% (13.4-17.2) | 8.3% (-7.4-24.0) |
| Male outcome, female exposure | 5.3% (5.1-5.6) | 9.2% (5.3-13.1) | 24.2% (21.3-27.2) | 27.1% (17.9-36.3) | 5.3% (5.1-5.6) | 9.2% (5.3-13.2) | 24.3% (21.3-27.3) | 27.1% (17.9-36.2) |
| Male outcome, male exposure | 4.9% (4.7-5.2) | 0.0% (0.0-0.0) | 24.3% (22.0-26.7) | 27.3% (-5.5-60.1) | 4.9% (4.7-5.2) | 0.0% (0.0-0.0) | 24.4% (22.0-26.7) | 27.2% (-3.9-58.2) |

Notes: 95% CI, 95% confidence intervals.
^1^ Adjusted for covariates sex, sex of relative, birth year, birth year of relative, birth order, and birth order of relative, where applicable. Proportions are standardized over covariates.

**Supplemental Table 2.** Odds ratio of a BPD diagnosis when having an ADHD diagnosis oneself, or a relative diagnosed with ADHD.

|  | **No. of individuals** | **Crude Odds Ratio**  **(95% CI)** | **Sex adjusted Odds Ratio**^1^  **(95% CI)** | **Birth year adjusted Odds Ratio**^2^  **(95% CI)** | **Birth order adjusted Odds Ratio**^3^  **(95% CI)** | **Adjusted Odds Ratio**^4^  **(95% CI)** |
| --- | --- | --- | --- | --- | --- | --- |
| **Within individual**^5^ | 2 113 902 | 11.4 (10.9-11.9) | 14.6 (14.0,15.3) | 16.1 (15.4,16.9) | 11.3 (10.9,11.9) | 19.4 (18.6-20.4) |
| **Relatives** | **No. of pairs**^6^ |  |  |  |  |  |
| Monozygotic twins | 9 130 | 5.0 (1.5-16.9) | 6.7 (2.0,22.9) | 10.5 (3.0,36.4) | 4.9 (1.4,16.7) | 11.2 (3.0-42.2) |
| Dizygotic twins | 17 350 | 0.6 (0.1-4.7) | 0.7 (0.1,4.7) | 1.1 (0.1,7.7) | 0.6 (0.1,4.5) | 1.0 (0.1-7.3) |
| Full siblings | 2 211 396 | 2.4 (2.2-2.6) | 2.4 (2.2,2.6) | 2.9 (2.6,3.1) | 2.3 (2.1,2.5) | 2.8 (2.6-3.1) |
| Maternal half-siblings | 332 486 | 1.4 (1.2-1.6) | 1.4 (1.2,1.6) | 1.4 (1.2,1.7) | 1.3 (1.1,1.5) | 1.4 (1.2-1.7) |
| Paternal half-siblings | 331 080 | 1.5 (1.3-1.6) | 1.5 (1.3,1.7) | 1.5 (1.3,1.7) | 1.4 (1.3,1.6) | 1.5 (1.3-1.7) |
| Cousins parents full-siblings | 6 456 848 | 1.4 (1.3-1.5) | 1.4 (1.3,1.5) | 1.5 (1.4,1.6) | 1.4 (1.3,1.5) | 1.5 (1.4-1.6) |
| Cousins parents maternal half-siblings | 472 212 | 1.2 (1.0-1.4) | 1.2 (1.0,1.4) | 1.3 (1.1,1.5) | 1.2 (1.0,1.4) | 1.3 (1.1-1.5) |
| Cousins parents paternal half-siblings | 466 836 | 1.2 (1.0-1.4) | 1.2 (1.0,1.4) | 1.2 (1.0,1.4) | 1.2 (1.0,1.4) | 1.2 (1.0-1.4) |

Notes: 95% CI, 95% confidence intervals.

^1^ Adjusted for sex and sex of relative, wherever applicable.

^2^ Adjusted for birth year and birth year of relative, wherever applicable.

^3^ Adjusted for birth order and birth order of relative, wherever applicable.

^4^ Adjusted for sex, sex of relative, birth year, birth year of relative, birth order, and birth order of relative, wherever applicable.
^5^ Cluster-robust standard errors based on mothers as clusters.
^6^ Number of unique ways of combining pairs, i.e., a pair may be included twice, first with A as outcome person and B as exposure person, then with B as outcome person and A as exposure person.

**Supplemental Table 3.** Odds ratio of a BPD diagnosis when having an ADHD diagnosis oneself, or a relative diagnosed with ADHD. Sensitivity analysis with both ADHD and BPD defined more restrictively; follow-up complete (no missing data) between ages 7 and 12 (ADHD) and/or ages 18 and 23 (BPD). Total sample and sex-specific analyses.

| **Supplemental Table 3a: Total sample** |  |  |  |
| --- | --- | --- | --- |
|  | **No. of individuals** | **Crude Odds Ratio**  **(95% CI)** | **Adjusted Odds Ratio**^1^  **(95% CI)** |
| Within individuals^2^ | 216 956 | 12.3 (10.8-13.9) | 15.2 (13.3-17.3) |
| **Relatives** | **No. of pairs**^3^ |  |  |
| Monozygotic twins | 602 | NA | NA |
| Dizygotic twins | 613 | NA | NA |
| Full-siblings | 418 559 | 2.6 (2.3-3.0) | 2.6 (2.3-3.0) |
| Maternal half-siblings | 111 615 | 1.5 (1.2-1.7) | 1.5 (1.2-1.7) |
| Paternal half-siblings | 113 883 | 1.6 (1.4-1.9) | 1.7 (1.4-2.0) |
| Cousins parents full-siblings | 1 663 143 | 1.4 (1.3-1.6) | 1.4 (1.3-1.5) |
| Cousins parents maternal half-siblings | 135 423 | 1.3 (1.1-1.6) | 1.4 (1.1-1.6) |
| Cousins parents paternal half-siblings | 138 533 | 1.2 (1.0-1.5) | 1.2 (1.0-1.5) |

| **Supplemental Table 3b: Sex-specific analyses** |  |  |  |  |
| --- | --- | --- | --- | --- |
|  | **No. of individuals** | **Crude Odds Ratio**  **(95% CI)** | **Adjusted Odds Ratio**^1^  **(95% CI)** | **p-value**^4^ |
| Within females^2^ | 105 169 | 15.1 (13.1-17.3) | 15.0 (13.1-17.3) | 0.720 |
| Within males^2^ | 111 787 | 16.2 (11.1-23.6) | 16.2 (11.1-23.7) |  |
| **Full siblings** | **No. of pairs**^3^ |  |  |  |
| Female outcome female exposure | 97 971 | 2.6 (2.1-3.3) | 2.6 (2.1-3.3) | 0.984 |
| Female outcome male exposure | 103 268 | 2.5 (2.1-3.1) | 2.6 (2.2-3.1) |  |
| Male outcome female exposure | 110 881 | 2.5 (1.5-4.4) | 2.6 (1.5-4.5) | 0.512 |
| Male outcome male exposure | 106 439 | 3.3 (1.9-5.8) | 3.3 (1.9-5.8) |  |

Notes: 95% CI, 95% confidence intervals; NA, not applicable – too few observed diagnoses to calculate informative estimates. ^1^ Adjusted for covariates sex, sex of relative, birth year, birth year of relative, birth order, and birth order of relative, where applicable.
^2^ Cluster-robust standard errors based on mothers as clusters.
^3^ Number of unique ways of combining pairs, ie, a pair may be included twice, first with A as outcome person and B as exposure person, then with B as outcome person and A as exposure person.
^4^ For pairwise comparison of adjusted estimates.

**Supplemental Table 4**. Descriptive information using more restrictive definitions of ADHD and BPD; follow-up complete (no missing data) between ages 7 and 12 (ADHD) and/or ages 18 and 23 (BPD).

|  | **Sample with non-missing ADHD**  **(column percent)** | **ADHD**  **(percent)** | **Sample with non-missing BPD**  **(column percent)** | **BPD**  **(percent)** | **Sample with non-missing ADHD and BPD**  **(column percent)** | **ADHD**  **(percent)** | **BPD**  **(percent)** | **ADHD & BPD**  **(percent)** |
| --- | --- | --- | --- | --- | --- | --- | --- | --- |
| **Sample size** | 1 124 579 (100.0) | 57 837 (5.1%) | 1 175 155 (100.0) | 8 289 (0.7) | 216 956 (100.0) | 8 381 (3.9) | 1 124 (0.5) | 361 (0.2) |
| **Covariates** |  |  |  |  |  |  |  |  |
| **Sex** |  |  |  |  |  |  |  |  |
| Female | 549 007 (48.8) | 20 155 (3.7) | 570 396 (48.5) | 7 151 (1.3) | 105 169 (48.5) | 3 311 (3.1) | 1 013 (1.0) | 313 (0.3) |
| Male | 575 572 (51.2) | 37 682 (6.5) | 604 759 (51.5) | 1 138 (0.2) | 111 787 (51.5) | 5 070 (4.5) | 111 (0.1) | 48 (0.0) |

**Supplemental Table 5.** Odds ratio of a BPD diagnosis when having an ADHD diagnosis oneself, or a full sibling diagnosed with ADHD. Sensitivity analysis with ADHD including the Attention deficit disorder without hyperactivity diagnosis (ICD-10 code F98.8). Within individual and full sibling analyses.

|  | **No. of individuals** | **Crude Odds Ratio**  **(95% CI)** | **Adjusted Odds Ratio**^1^  **(95% CI)** |
| --- | --- | --- | --- |
| Within individuals^2^ | 2 113 902 | 11.4 (10.9-11.9) | 19.4 (18.5-20.4) |
| **Relatives** | **No. of pairs** |  |  |
| Full-siblings | 2 211 396 | 2.4 (2.2-2.6) | 2.8 (2.6-3.1) |

Notes: 95% CI, 95% confidence intervals. ^1^ Adjusted for covariates sex, sex of relative, birth year, birth year of relative, birth order, and birth order of relative, where applicable.
^2^ Cluster-robust standard errors based on mothers as clusters.
